# Real-Time Estimation for Roll Angle of Spinning Projectile Based on Phase-Locked Loop on Signals from Single-Axis Magnetometer

**DOI:** 10.3390/s19040839

**Published:** 2019-02-18

**Authors:** Zhaowei Deng, Qiang Shen, Zilong Deng, Jisi Cheng

**Affiliations:** School of Mechatronical Engineering, Beijing Institute of Technology, Beijing 100081, China; 2220170072@bit.edu.cn (Z.D.); 2120170199@bit.edu.cn (Z.D.); 2120160189@bit.edu.cn (J.C.)

**Keywords:** roll angle measurement, spinning projectile, 2-D CCF, magnetic sensor, FLL-assisted PLL

## Abstract

As roll angle measurement is essential for two-dimensional course correction fuze (2-D CCF) technology, a real-time estimation of roll angle of spinning projectile by single-axis magnetometer is studied. Based on the measurement model, a second-order frequency-locked loop (FLL)-assisted third-order phase-locked loop (PLL) is designed to obtain rolling information from magnetic signals, which is less dependent on the amplitude and able to reduce effect from geomagnetic blind area. Method of parameters optimization of tracking loop is discussed in the circumstance of different speed and it is verified by six degrees of freedom (six degrees of freedom (DoF)) trajectory. Also, the measurement error is analyzed to improve the accuracy of designed system. At last, experiments on rotary table are carried out to validate the proposed method indicating the designed system is able to track both phase and speed accurately and stably. The standard deviation (SD) of phase error is no more than 3°.

## 1. Introduction

The research on smart ammunitions has long been a popular subject for the conventional uncontrolled projectiles cannot satisfy the requirements of modern warfare, high efficiency, accuracy, as well as low cost and collateral damage [1,2]. Thus, recent decades have witnessed the emergence of various intelligent weapons, such as terminal sensitive projectile [3], precision-guided munition [4], loitering munition [5], networked munition [6], intelligent munitions system [7], and trajectory correction projectile [8].

Among these developed technologies, the trajectory correction projectile is based on the transformation of conventional spinning projectiles through trajectory correction fuze (CCF). It can not only improve the precision but also be of great significance for destocking of dumb ones. There are two types of CCF: one-dimension CCF (1-D CCF) [9] and two-dimension CCF (2-D CCF) [10]. The technology of 1D-CCF (omnidirectional antenna is applied in the Global Navigation Satellite System (GNSS) receivers of 1-D CCF to obtain the position and velocity) is now mature enough to be equipped in the army force worldwide, but has limited precision for correcting the course in the longitudinal direction only.

2-D CCF minimizes impact point dispersion better than 1-D CCF because it is able to correct both longitudinal and lateral errors by fixed canard; the real-time estimation of the spinning projectile roll angle is key to control the fixed canards to provide deflection command.

However, under the firing circumstance of high-g (≥ 12,000 g) and high spin (≥ 10 Hz), it is a challenge for sensors to measure the spinning information, roll angle, and rotational speed. There are several researches focusing on this daunting task for a long time. Park and Kim [11] and Harkins and Wilson [12] attempted to solve this problem by inertial instruments such as gyroscopes but results showed it works properly only within low-dynamic range.

The GNSS and magnetoresistive sensor seem now the best choice to determine the roll information for they can resist the harsh gun-launching environment. Some corporations and institutes have focused on the technology on single-patch antennas mounted on the side of a spinning vehicle to get both location and rotary information. Shen, Li, and Deng studied a method to reach that by tracking discontinuous signals from their designed antenna [13,14], however it was not mature and stable enough resulting in occasional bad measurements in location and velocity and the tracking loop, first-order FLL-assisted second-order PLL designed by Deng and Shen limits in tracking signals phase of which changing in the form of ramp function. On the other hand, the magnetometer owns the advantages of passive sensing, high sensitivity, as well as low power and cost. Thus, the research on measurement based on magnetic sensor has always prospered. The French-German Research Institute of Saint-Louis (ISL) studied on obtaining rolling information of an air defense projectile by two embedded magnetometers [15] and attitude estimation of projectiles using magnetometers and accelerometers [16]. U.S. Army Research Laboratory (ARL) put forward kinds of integrated navigation based on magnetometers. POINTER system [17] is the combination of SOLARSONDE and MAGSONDE consisting of solar sensors and magnetic sensor. Another attitude determination system [18] used three magnetometers aligned with body coordinate system assisted by angular rate sensors to figure out all three Euler angles. ARL achieved accuracy within 5° in roll angle measurement and ISL even did better in experimental validation [19] after compensation. However, in all these research above, the precision is affected by conventional algorithm of magnetic measurement, more than one magnetic sensor and based on the amplitude of magnetic sensors’ output. ARL has toiled and moiled in lab experimental validation to make precision better for years by extended Kalman filter (EKF). Wang and Cao have done lots of work in filter sensor calibration and curve fitting to eliminate errors resulting from the conventional method [20,21]. Wang reached an acceptable accuracy of roll angle measurement within 5° but the compensation complicates the system and results in increased power consumption. To overcome the weakness of traditional method based on geomagnetism, some institutes presented methods based on time–frequency domain analysis [22] and frequency-locked loop [23] information obtained from single-axis magnetic sensor rather than multiple outputs or operation of inverse trigonometric function. They managed to measure the rotational speed but no progress in roll angle. Additionally, all these study above did not explain clearly how to address the problem of geomagnetic blind area [22]. Zhang proposed the conception of blind area of roll angle, certain range of roll angle in which detection module cannot satisfy the expected accuracy [24], which will be recapped to assist the method proposed in this paper to reduce the effect from blind area.

Inspired by the integrated navigation and advantages from both GNSS and magnetoresistive sensors, a real-time estimation for roll angle based on phase-lock loop on signals from single-axis magnetoresistive is proposed. Unlike the conventional measurement by an inverse trigonometric operation based on amplitude of multiple outputs, a second-order frequency-locked loop (FLL)-assisted third-order phase-locked loop (PLL) is designed to obtain the phase information of the output of single-axis magnetometer, which is relevant to rolling attitude. Meanwhile, angle of pitch and yaw obtained from the omnidirectional antenna (technology of 1D-CCF), and declination, inclination obtained from IGRF (International Geomagnetic Reference Field) model [25], the real-time rotational information can be figured out. It is able to avoid the measurement error from conventional ways and reduce effect from blind area, reaching cost-reduction, low-power consumption, as well as miniaturation.

This novel method is proposed to obtain rolling attitude of kinds of spin stabilized projectiles launched by howitzer or tank. They have a frequency range from 3 Hz to 300 Hz. Also, it could be applied to measure the roll angle of vehicle in the state of continuous rotation.

In the following sections, at first, measurement model is established after the description of geomagnetic field vector in different coordinate systems. Then, the method based on second-order FLL-assisted third-order PLL to detect the rotatory attitude is studied and optimized. After that, error analysis of measurement model is discussed in detail. Solutions are studied to address different errors including that resulted from tracking system, compensation angle (defined in chapter 2), blind area. At last, the results of experiments on rotary table show the system designed is able to work effectively with a good accuracy.

## 2. Measurement System Modeling

Description of different coordinate systems is necessary for the mathematical model of measurement. In this section, transformations are explained and then the measurement system is modeled.

### 2.1. Description of Geomagnetic Vector in a North-East-Down Coordinate System

**Figure 1:**
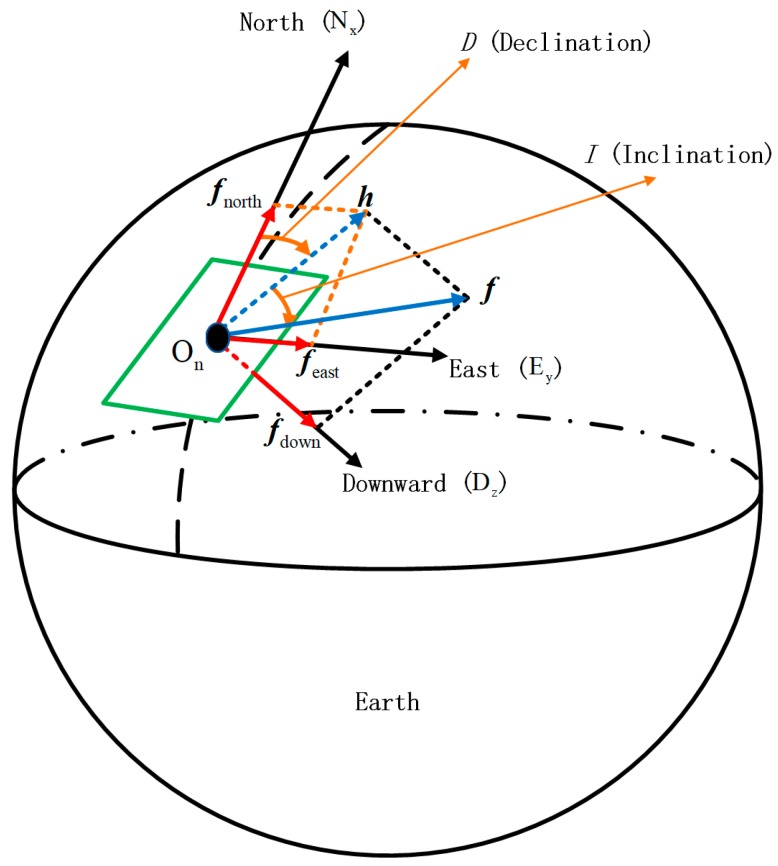
Description of geomagnetic field.

As shown in Figure 1, the local North-East-Down (NED) coordinate system is the coordinate frame to describe the geomagnetic vector and fixed to the surface of earth. The origin denoted by $O_{n}$ is arbitrarily fixed to a point on the surface of earth, the X-axis denoted by $N_{x}$ points to the geodetic north, the Y-axis is denoted by $E_{y}$ points to the geodetic east, and the Z-axis is denoted by $D_{z}$ points downward along the ellipsoid normal [26].

As shown in the Figure 1, elements to describe the field are as follows.

(1) $$\left\{ \begin{array}{l} \left|h\right|=\left|f\right|\cos I\\ \left|f_{\mathrm{down}}\right|=\left|f\right|\sin I\\ \left|f_{\mathrm{north}}\right|=\left|h\right|\cos D=\left|f\right|\cos I\cos D\\ \left|f_{\mathrm{east}}\right|=\left|h\right|\sin D=\left|f\right|\cos I\sin D\\ f^{2}=h^{2}+f^{2}{}_{\mathrm{down}}=f^{2}{}_{\mathrm{north}}+f^{2}{}_{\mathrm{east}}+f^{2}{}_{\mathrm{down}} \end{array}\right.$$

where, f is the geomagnetic vector and |f| is the total field intensity; h is horizontal component of total vector; D is declination, the angle between h and north, and positive when east; I is inclination, the angle between total vector f and horizontal plane, positive when downward; $f_{\mathrm{north}}$ is projection of f on north direction; $f_{\mathrm{east}}$ is projection of f on east direction; $f_{\mathrm{down}}$ is projection of f on downward direction.

According to latitude, longitude, and elevation of the practical location, all components above relative to the geomagnetic information can be obtained from the World Magnetic Model (WMM) [27] and the International Geomagnetic Reference Field (IGRF) model. 

### 2.2. Coordinate Transforamtions

**Figure 2:**
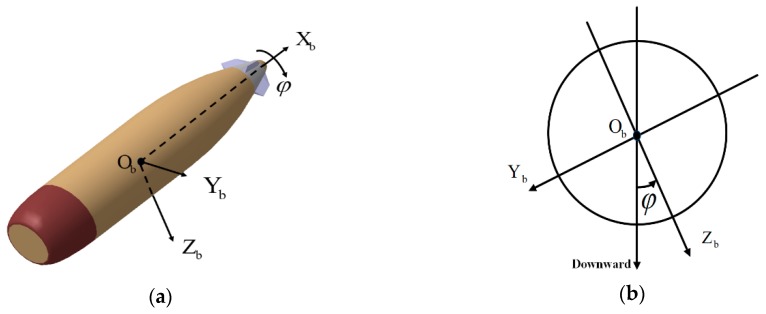
Here are figures to describe projectile body based on a model of 155 mm artillery projectile launched by howitzer: (**a**) Description of body coordinate system and (**b**) description of the cross-section.

Description of body coordinate system is necessary for the transformation. As shown in Figure 2a, the body coordinate system, directly defined on the flying projectile, whose origin is denoted by $O_{b}$, locates at the center of gravity of the flying vehicle; the X-axis, denoted by $X_{b}$, points forward to the head of body; the Y-axis, denoted by $Y_{b}$, points to the right side of the body and perpendicular to the symmetric plane; and the Z-axis, denoted by $Z_{b}$, points downward to comply the right-hand rule. Figure 2b shows the cross-section of projectile.

Define ψ, θ, φ, as yaw, pitch, and roll angle respectively, and $C_{n}^{b}$ as the transformation matrix from NED to body coordinate system, according to Figure 1 and Figure 2:

(2) $$C_{n}^{b}=\left[\begin{array}{ccc} 1 & 0 & 0\\ 0 & \cos\varphi & \sin\varphi \\ 0 & -\sin\varphi & \cos\varphi \end{array}\right]\left[\begin{array}{ccc} \cos\theta & 0 & -\sin\theta \\ 0 & 1 & 0\\ \sin\theta & 0 & \cos\theta \end{array}\right]\left[\begin{array}{ccc} \cos\psi & \sin\psi & 0\\ -\sin\psi & \cos\psi & 0\\ 0 & 0 & 1 \end{array}\right]$$

### 2.3. Mathematical Model

${\left[\begin{array}{ccc} \left|f_{\mathrm{north}}\right| & \left|f_{\mathrm{east}}\right| & \left|f_{\mathrm{down}}\right| \end{array}\right]}^{T}$ (superscript T indicates transposition) is the projection of geomagnetic vector into NED system, while ${\left[\begin{array}{ccc} B_{X} & B_{Y} & B_{Z} \end{array}\right]}^{T}$ is the projection onto body coordinate system. According to (3), they can be expressed as follows

(3) $$\left[\begin{array}{c} B_{X}\\ B_{Y}\\ B_{Z} \end{array}\right]=\left[\begin{array}{ccc} \cos\psi \cos\theta & \cos\theta \sin\psi & -\sin\theta \\ \cos\psi \sin\varphi \sin\theta -\cos\varphi \sin\psi & \cos\varphi \cos\psi +\sin\gamma \sin\psi \sin\theta & \cos\theta \sin\varphi \\ \sin\varphi \sin\psi +\cos\varphi \cos\psi \sin\theta & \cos\varphi \sin\psi \sin\theta -\cos\psi \sin\varphi & \cos\varphi \cos\theta \end{array}\right]\left[\begin{array}{c} \left|f_{\mathrm{north}}\right|\\ \left|f_{\mathrm{east}}\right|\\ \left|f_{\mathrm{down}}\right| \end{array}\right]$$

From the analysis on (3), the mathematical relationship between roll angle and projection of geomagnetic vector into cross-section of projectile is presented as

(4) $$B_{Z}=\sqrt{w^{2}+v^{2}}\sin(\varphi +\varepsilon )$$

where,

(5) $$w=\left|f_{\mathrm{north}}\right|\sin\psi -\left|f_{\mathrm{east}}\right|\cos\psi$$

(6) $$v=\left|f_{\mathrm{north}}\right|\cos\psi \sin\theta +\left|f_{\mathrm{east}}\right|\sin\psi \sin\theta +\left|f_{\mathrm{down}}\right|\cos\theta$$

(7) $$\varepsilon =\{\begin{array}{l} \arctan\left|\frac{v}{w}\right|,\;\;v\geq 0,w\geq 0\\ 2\pi -\arctan\left|\frac{v}{w}\right|,\;\;v\geq 0,w<0\\ \pi -\arctan\left|\frac{v}{w}\right|,\;\;v<0,w\geq 0\\ \pi +\arctan\left|\frac{v}{w}\right|,\;\;v<0,w<0 \end{array}$$

In Equation (4), ε is the compensation angle, and it is the difference between phase angle of $B_{Z}$ and φ. Moreover, it is irrelevant with total field intensity |f| but depends on the D, I and ψ, θ.

When the rotation reverses, φ=−φ and ε=π−ε, Equation (4) is presented as

(8) $$B_{Z}=\sqrt{w^{2}+v^{2}}\sin(-\varphi +(\pi -\varepsilon ))$$

Similarly, one can also get the relationship between roll angle and $B_{Y}$ from (3) through the derivation above:

(9) $$B_{Y}=\sqrt{w_{1}{}^{2}+v_{1}{}^{2}}\sin(\varphi +\varepsilon_{1})$$

**Figure 3:**
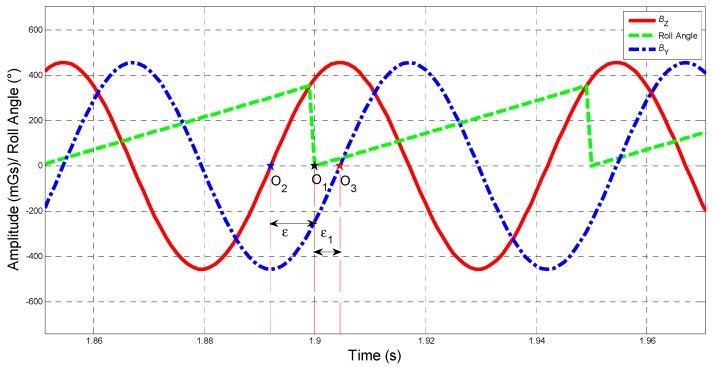
Description of compensation angle.

When the total field is 500 mGs, declination is 59.263°, inclination is −6.8285°, pitch is 18°, yaw is 100°, and rotational speed is 20 Hz, the sinusoidal signals and roll angle are shown in Figure 3.

Shown in Figure 3, the time difference between $O_{1}$ and $O_{2}$, as well as time difference between $O_{1}$ and $O_{3}$, indicates the compensation angle ε$(\varepsilon_{1}),$ constant difference between the phase of $B_{Z}$$\left(B_{Y}\right)$ and the roll angle.

To make a conclusion, if one manages to extract the phase information of $B_{Z}$ (or $B_{Y})$ in (4) (or (9)) and figure out the compensation angle ε (or $\varepsilon_{1})$ which is independent on the amplitude of $B_{Z}$ ($B_{Y})$ or the total intensity |f|, the roll angle φ will be obtained.

## 3. Method to Obtain the Rolling Information

From analysis above, it is essential for obtaining roll angle to figure out the phase information of $B_{Z}.$ In this section, a tracking loop and frequency-locked loop (FLL)-assisted phase-locked loop (PLL) are designed to track the information necessary of magnetic signals.

**Figure 4:**
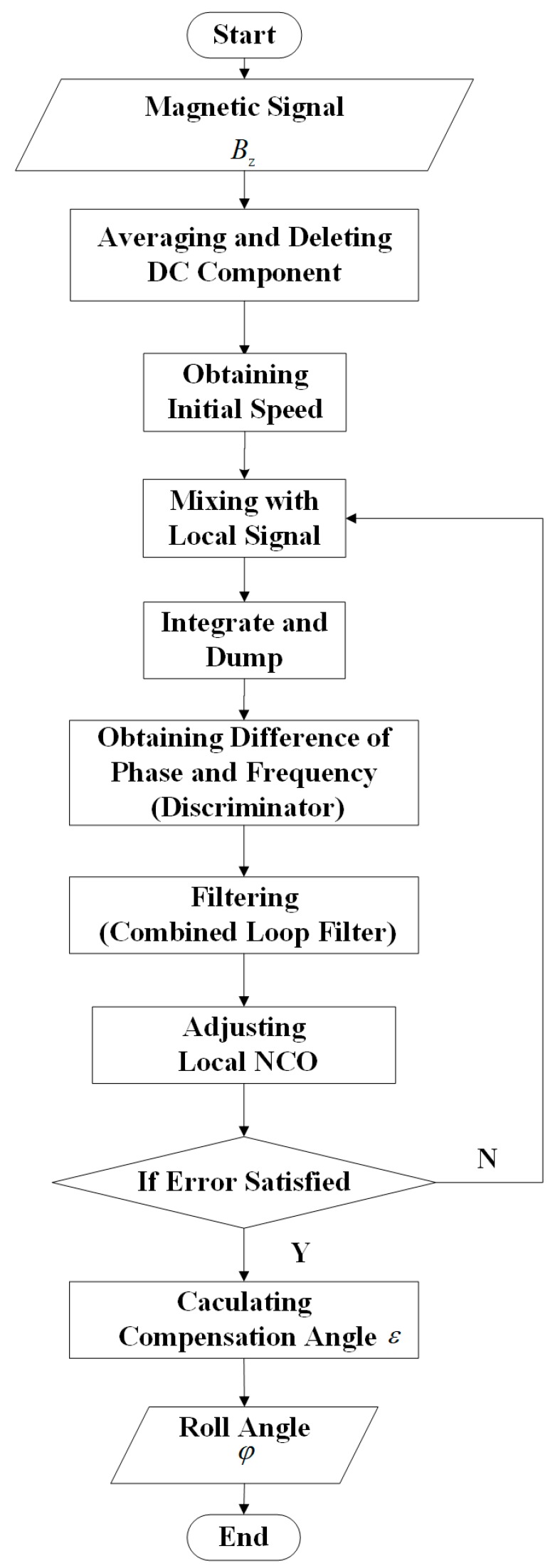
Process of obtaining roll angle.

Inspired by Deng and Shen proposing a first-order FLL-assisted second-order PLL to track GPS signals received by a single-patch antenna [14], a combined loop filter, second-order FLL-assisted third-order PLL is designed to track magnetic signals for the spinning projectile rotates in the form of acceleration function (frequency changes in the form of ramp function). The whole process of obtaining roll angle from spinning projectile is shown in Figure 4. 

In practice, initial speed obtained by FFT (fast Fourier transform) at the beginning of signals tracking is set as the initial frequency for NCO.

To optimize parameters of tracking loop to fit perfectly to kinds of range of rolling speed, amplitude–frequency response, transient response, and analysis of integration time are presented in following work. Then, the pitch, yaw, and rolling speed, based on six degrees of freedom (six DoF) trajectory, are set as the input of the tracking loop to test the system and analyze the performance.

### 3.1. Design of Tracking Loop

The phase-locked loop (PLL) is a kind of closed-loop control system. It obtains the information about the phase and frequency of the input through a numerically controlled oscillator (NCO) creating synchronous output [28]. In chapter 2, measurement model indicates that with the rotation of spinning projectile, the geomagnetic vector projecting on cross-section $\left(B_{Z}\right)$ changes in the form of sinewave, which is the input of PLL. PLL achieves the phase information of input by obtaining the frequency and phase of local signal from NCO.

**Figure 5:**
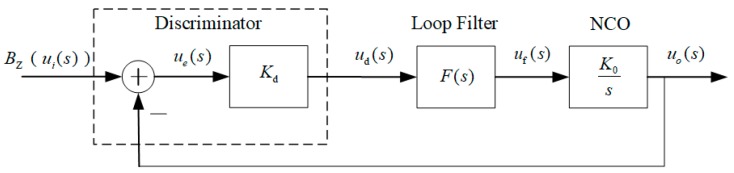
Block diagram of phase-locked loop in frequency domain (Laplace transform).

As shown in Figure 5, PLL consists of three parts, discriminator, loop filter, and NCO. It shows the output of NCO is fed back to the front, forming a closed loop control system. It also can be presented as the block diagram of FLL for the only difference between FLL and PLL is the discriminator, where arctangent discriminator [29] is applied in PLL to obtain the difference of phase, and cross-product frequency tracking [29] is applied in FLL to obtain the difference of frequency.

$u_{i}(s)$ and $u_{o}(s)$ are the Laplace transformations of input $B_{Z}$ and the output, respectively; $u_{e}(s)$ obtained by discriminator is the difference between $u_{i}(s)$ and $u_{o}(s);$ $K_{d}$ is the gain of discriminator; $u_{d}(s)$ is the output of discriminator; F(s) is a loop filter, the output of which is $u_{f}(s);$$\frac{K_{o}}{s}$ is the Laplace transformation of NCO. Thus, transfer function of above system can be expressed as

(10) $$H(s)=\frac{u_{o}(s)}{u_{i}(s)}=\frac{K_{o}K_{d}F(s)}{s+K_{o}K_{d}F(s)}=\frac{KF(s)}{s+KF(s)}$$

where, F(s) is the transfer function of loop filter, $K_{d}$ and $K_{o}$ are the gain of discriminator and NCO, respectively. The gain of loop filter can be expressed as

(11) $$K=K_{o}K_{d}$$

PLL is able to track the target signals with low noise precisely, but the narrow bandwidth restricts its accuracy in high-dynamic situations. Furthermore, it does not work properly when tracking signals are with much noise. In contrasts, the FLL owns a comparatively wide bandwidth and good dynamic performance. However, FLL is applied to track frequency of signal and has less efficiency in obtaining phase information. To satisfy both accuracy and dynamic performance, a FLL-assisted PLL loop combining advantages of PLL and PLL was designed to track the target signals quickly and accurately.

**Figure 6:**
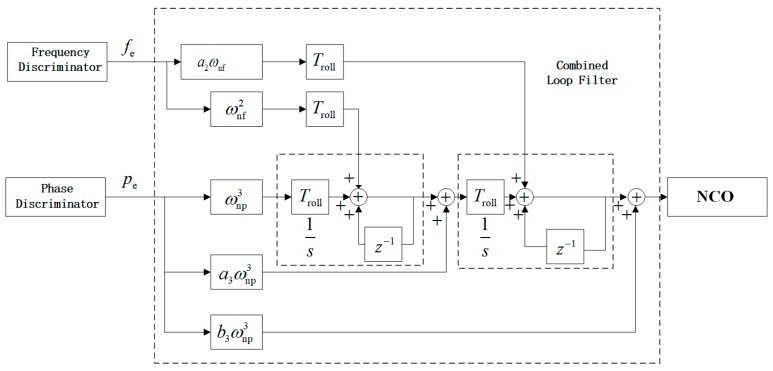
Block diagram of discrete time system of combined loop filter.

What is shown in Figure 6 is the discrete time system of second-order FLL-assisted third-order PLL. The combined loop filter is the Z transform of F(s) in Figure 5, which consists of $F_{p}(s)$, a transfer function of third-order PLL, as well as $F_{f}(s)$, transfer function of second-order FLL. Both frequency discriminator and phase discriminator in Figure 6 make up discriminator in Figure 5. $p_{e}$ and $f_{e}$ are the outputs of discriminators input to second-order FLL and third-order PLL, respectively.

**Figure 7:**
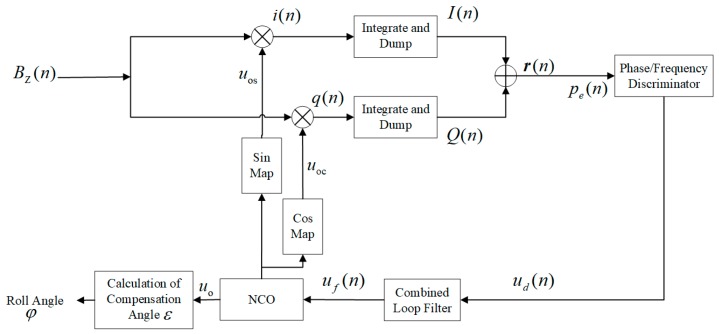
Designed tracking system.

There are parameters to be determined to optimize system to keep good performance in different situations, which are $T_{\mathrm{roll}}$, unit delay (it is also the integration time of integrate and dump process in Figure 7 and discussed in chapter 4), damping ration ξ ($a_{2}=2\xi$), natural frequency $\omega_{\mathrm{nf}}$ of second-order FLL as well as natural frequency $\omega_{\mathrm{np}}$ of third-order PLL. Details of the selection of parameters $a_{3}$ and $b_{3}$ can be found in “Understanding GPS: principles and ap plications, 2nd Ed” [29].

According to Figure 6, transfer functions of filter loops of third-order PLL and second-order FLL can be expressed as

(12) $$F_{p}(s)=\omega_{\mathrm{np}}^{3}\frac{1}{s^{2}}+a_{3}\omega_{\mathrm{np}}^{2}\frac{1}{s}+b_{3}\omega_{np}$$

(13) $$F_{f}(s)=2\xi \omega_{\mathrm{nf}}+\frac{\omega_{\mathrm{nf}}^{2}}{s}$$

According to Figure 5 and equation (10), the transfer function of the third-order PLL and second-order FLL can be expressed as

(14) $$H_{p}(s)=\frac{b_{3}\omega_{\mathrm{np}}s^{2}+a_{3}\omega_{\mathrm{np}}^{2}s+\omega_{\mathrm{np}}^{3}}{s^{3}+b_{3}\omega_{\mathrm{np}}s^{2}+a_{3}\omega_{\mathrm{np}}^{2}s+\omega_{\mathrm{np}}^{3}}$$

(15) $$H_{f}(s)=\frac{2\xi \omega_{\mathrm{nf}}s+\omega_{\mathrm{nf}}^{2}}{s^{2}+2\xi \omega_{\mathrm{nf}}s+\omega_{\mathrm{nf}}^{2}}$$

According to Figure 5 and Figure 6, the diagram of discrete-time tracking system can be described as Figure 7:

As shown in Figure 7, the I/Q Demodulator [14,29] is applied in the Costas Loop to assist discriminators to obtain the difference of phase and frequency ($p_{e}$ and $f_{e}$) between input and output. $B_{z}$ is the input of tracking system which exports $u_{o}$. Phase information obtained by reading $u_{o}$, then with calculation of compensation angle ε, φ can be figured out. 

### 3.2. Analysis of Performance and Parameters Optimized

Obviously, analysis of performance and parameters optimization starts from the premise that tracking system is capable of tracking signal originated from spinning projectile rotating in the form of acceleration function. Thus, analysis of steady-state error will be presented at first.

According to Figure 5, and (10), the difference between $u_{o}(s)$ and $u_{i}(s)$ is described as

(16) $$u_{e}(s)=\frac{s}{s+KF(s)}u_{i}(s)$$

when the object rotates in the form of acceleration function, $u_{i}(s)=\frac{k_{r}}{s^{3}}$($k_{r}$ is the change of rate in speed) and $F(s)=F_{P}(s).$ Referring to final-value theorem [30], the steady-state error $e_{\mathrm{ss}}(\infty )$ of $H_{\mathrm{pe}}(s)$ can be described as

(17) $$e_{\mathrm{ss}}(\infty )=\underset{s\to 0}{\lim}su_{e}(s)$$

thus,

(18) $$e_{\mathrm{ss}}(\infty )=\underset{s\to 0}{\lim}\frac{k_{r}s}{s^{3}+b_{3}\omega_{\mathrm{np}}s^{2}+a_{3}\omega_{\mathrm{np}}^{2}s+\omega_{\mathrm{np}}^{3}}=0$$

From analysis above, the designed third-order PLL is capable of tracking the phase signal changing in the form of acceleration function. Similarly, the designed second-order FLL is able to track the frequency signal which changes in the form of ramp function.

According to (14), the noise bandwidth of PLL, $B_{\mathrm{PLL}},$ is given by

(19) $$B_{\mathrm{PLL}}=\int_{0}^{\infty }{\left|H_{p}(j2\pi f)\right|}^{2}df=\frac{b_{3}^{2}a_{3}+a_{3}^{2}-b_{3}}{4(a_{3}b_{3}-1)}\omega_{\mathrm{np}}$$

where, f is the frequency and $H_{p}$ is the frequency response function of the third-order PLL. Referring to [29],

(20) $$b_{3}=2.4,\;\;a_{3}=1.1$$

**Figure 8:**
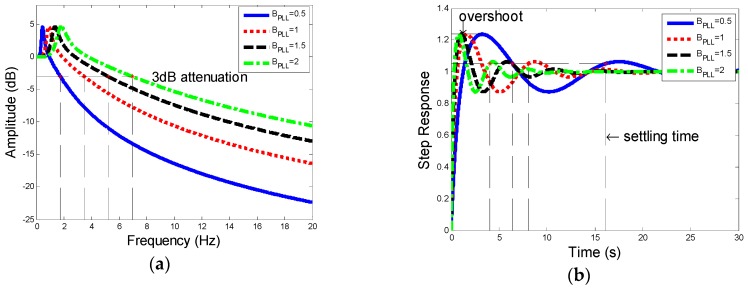
(**a**) Descriptions of amplitude–frequency characteristic. (**b**) Descriptions of step response of third-order PLL.

As shown in Figure 8, the amplitude–frequency characteristic and step response of PLL vary as $B_{\mathrm{PLL}}$ changes:

Figure 8 shows how noise bandwidth $B_{\mathrm{PLL}}$ influence amplitude–frequency and transient characteristics. Larger the bandwidth is, the better the transient performance is (shorter settling time). However, with a lager $B_{\mathrm{PLL}},$ it will have a worse amplitude–frequency characteristic and a greater cut-off frequency, which leads to a reduction of tracking accuracy.

**Figure 9:**
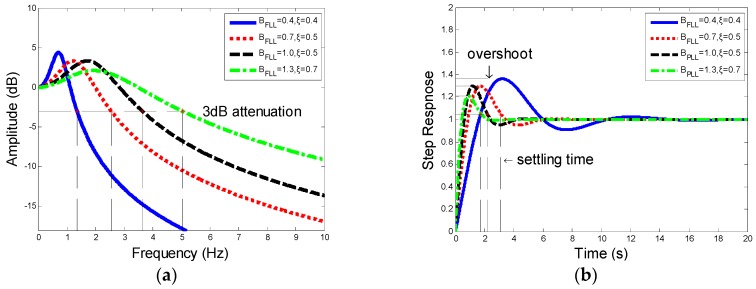
(**a**) Description of amplitude–frequency characteristic. (**b**) Description of step response of second-order FLL.

According to (15), the noise bandwidth of FLL, $B_{\mathrm{FLL}},$ is given by

(21) $$B_{\mathrm{FLL}}=\int_{0}^{\infty }{\left|H_{F}(j2\pi f)\right|}^{2}df=\frac{\omega_{\mathrm{nf}}}{2}\left(\xi +\frac{1}{4\xi }\right)$$

where, $H_{F}$ is the frequency response function of second-order FLL. The damping ratio ξ and noise bandwidth $B_{\mathrm{FLL}}$ both determine the frequency at the −3 dB point, settling time, and overshoot, which is shown in Figure 9.

Similar to PLL, the results of the amplitude–frequency and step response show that with the damping ratio ξ and noise bandwidth $B_{\mathrm{FLL}}$ increased, the cut-off frequency becomes larger, and the overshoot and settling time become shorter.

Influence of parameters on tracking system is shown in Table 1.

From the analysis above, one can make a conclusion that for the tracking system designed, the performance of amplitude–frequency contradicts the transient performance. For PLL, which ensures the tracking precision, amplitude–frequency performance plays a more important role. FLL in the combined tracking loop is designed to lock the frequency and pull the loop into phase-locking state as quickly as possible, therefore transient performance should be given priority.

Furthermore, choosing optimum damping ξ=0.707 would optimize the two-order FLL to the greatest extent. Meanwhile, the selected noise bandwidths $B_{\mathrm{PLL}}$ and $B_{\mathrm{FLL}}$ should fit to different rotational speed which ranges from 3 Hz to 300 Hz. For the low speed, the selected noise bandwidth must enable the frequency at −3 dB point to be less than 3 Hz and for a higher rotational speed the noise bandwidth should be improved depending on the practice.

Besides, the integration time $T_{\mathrm{roll}}$ in Figure 6 determines the accuracy of tracking to a large degree. Referring to [14,29], when $T_{\mathrm{roll}}$ is five times longer than the period T of input or set as integer multiple of the T of input affect caused by high-frequency components can be eliminated.

In conclusion, how to select the optimal parameters for the designed tracking loop depends on both theoretical analysis and practice. Parameters determining the accuracy and transient performance should be balanced in different rotational speed. In the state of low rotational speed, parameters should be adjusted to give priority to amplitude–frequency characteristic as well as steady-state performance. The higher the rotational speed is, the more important the transient performance is. What is more, selection of parameters in PLL and FLL varies. The former focuses on accuracy and another focuses on transient response. 

To verify the proposed method to optimize tracking system and show the details of how to select optimal parameters, a simulation based on 6-DoF trajectory is presented in the follows.

### 3.3. Model Verification Based on 6 DoF Trajectory

From the analysis above, the design and optimization of the tracking system depends on the frequency of input $B_{Z}$ to a large extent. In practice, the rotational speed of a spinning projectile is determined by the type of projectile and launching platform. Meanwhile, information of geomagnetic field can be obtained from WMM and IGRF mentioned in chapter 2 and yaw and pitch are given by GPS receivers (or other sensors) in this proposed technology. In this simulation, a 6-DoF trajectory [31] would provide information needed to verify the designed tracking loop. 

**Figure 10:**
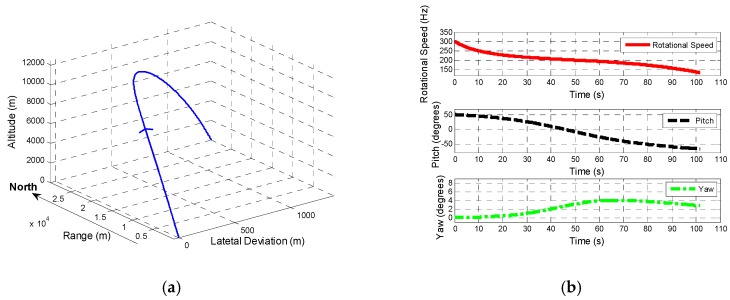
Model of 6-degrees of freedom (DoF) trajectory: (**a**) Trajectory of projectile and (**b**) information of the speed, pitch and yaw from 6-DoF.

**Figure 11:**
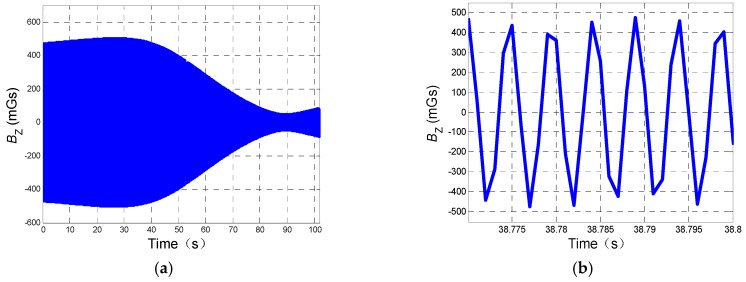
Amplitude of $B_{Z}$ changing in flight: (**a**) Description of the amplitude of $B_{Z}$. (**b**) Description of part of the amplitude of $B_{Z}$ (within 0.03 second).

Based on a 6-DoF trajectory, as shown in Figure 10a, of a 155 mm artillery projectile, the properties of which are listed in Table 2, the designed tracking loop is applied to obtain the roll angle of spinning projectile based on the information of yaw and pitch (obtained from GPS in practice) is shown in Figure 10b. Table 2 also provides the details of geomagnetic field (assuming that declination and inclination changes slightly in the range of howitzer). Figure 11a shows the change of amplitude of during flight, while Figure 11b shows the amplitude changing within 0.03 second.

From Figure 10b, yaw varies (from 0° to 4°) slighter than pitch (from 50° to −67°), and the rotational speed drops from 300 Hz to 134 Hz in the form of acceleration function.

Figure 11 indicates that in flight, amplitude of $B_{Z}$ changes in the form of sinewave along with the change of pitch and yaw.

Based on (14), (15), (20), and ξ=0.707, noise bandwidth—$B_{\mathrm{PLL}}$ and $B_{\mathrm{FLL}}$—are two parameters to be determined to optimize the 2-order FLL-assisted 3-order PLL tracking loop. From Figure 8 and Figure 9 it can be seen that the frequency at 3 dB attenuation increases with the raising of noise bandwidth. For the tracking accuracy, the cut-off frequency has to be not less than the lowest spinning speed (3Hz). However, with a higher range of rotational speed, the cut-off frequency can be improved slightly at the aim of a quick transient response especially for FLL. Thus, in the circumstance of spinning speed dropping from 300 Hz to 134 Hz, the $B_{\mathrm{PLL}}$ is set as 0.65 and $B_{\mathrm{FLL}}$ is set as 0.7, which also indicates that transient performance is prior to steady-state performance in FLL. Meanwhile, the period of input signal is from 3.33 ms to 7.46 ms. Based on the analysis, integration time $T_{\mathrm{roll}}$ should be five times more than T to ensure tracking accuracy, it is set as 40 ms. Therefore, based on the 6-DoF trajectory above, the optimized parameters are listed at Table 3.

After optimization of the designed system, the tacking results are given as follows.

**Figure 12:**
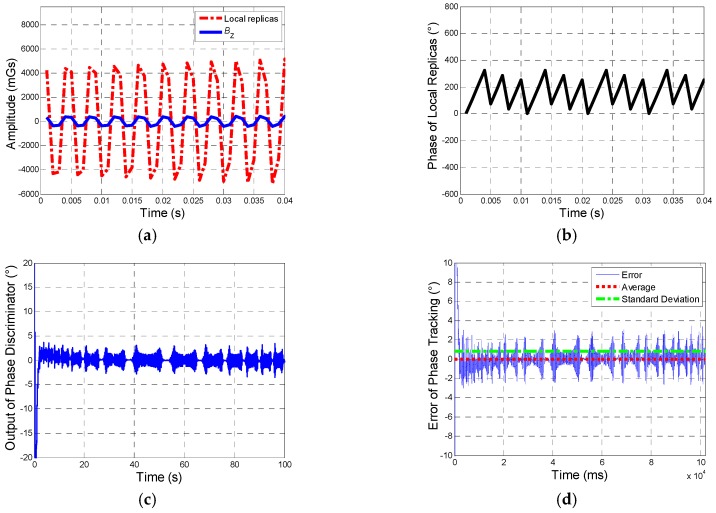
Tracking results. (**a**) Description of $B_{Z}$ and local replicas (amplitude of local replicas is amplified 8 times for the convenience of comparison). (**b**) Description of phase of local replicas. (**c**) Description of the output of phase discriminator. (**d**) Description of error of phase tracking.

Figure 12 shows the tracking results. There are amplitude of input $B_{Z}$ and output of tracking system, the phase of tracking signals, phase discriminator output, as well as error of phase tracking. (a) indicates that the output of tracking system tracks the input well. (b) shows that phase of output changes from 0° to 360°, which means the phase of $B_{Z}$ gets locked stably, and (d) describes the error of phase tracking, which proves an excellent tracking performance of system designed: phase error between input and output gets steady within 3 seconds and keeps within 3°, Average error is −0.0228° and standard deviation (SD) is 0.7868°, which indicates a stable phase tracking result.

**Figure 13:**
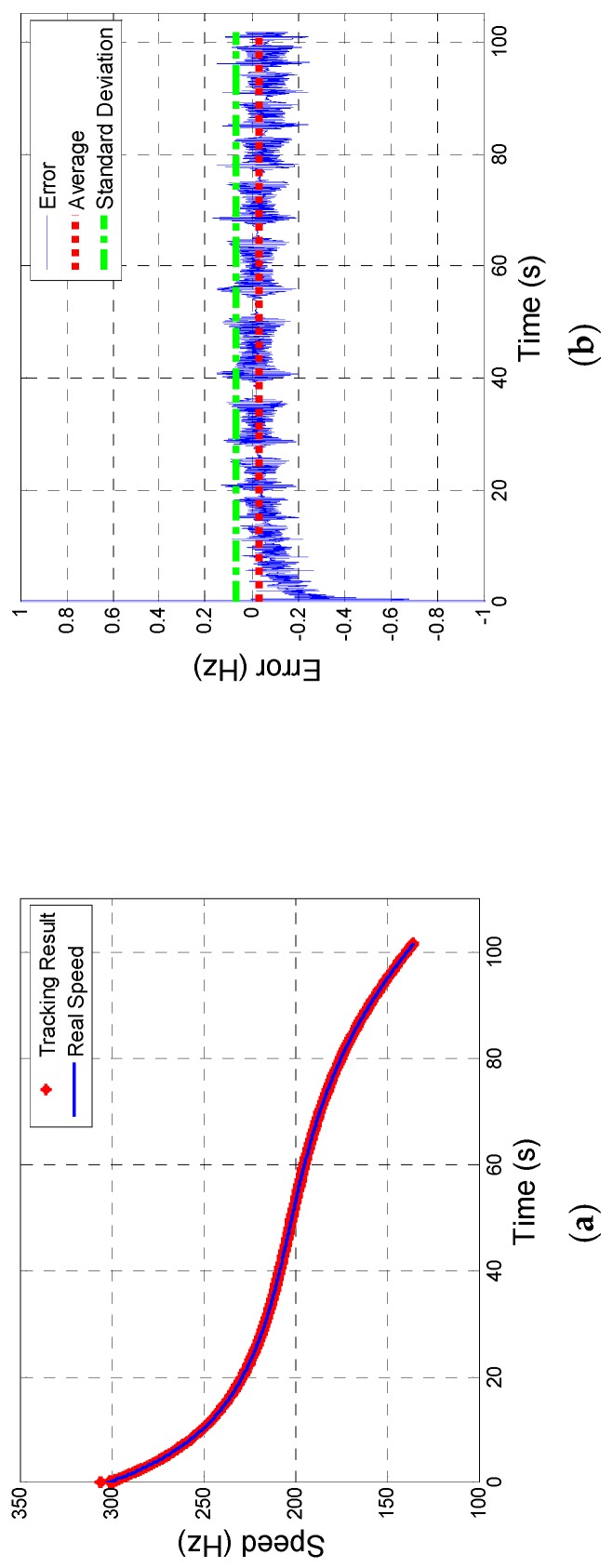
Tracking results: (**a**) Description of the contrast of speed and (**b**) description of error of speed tracking.

Figure 13 shows the tracking results of speed which verify a desirable speed tacking performance of designed system.

Figure 13a is the contrast between real one and tracking system and b indicates that error of speed tracking becomes steady after 10 seconds: average error is −0.034 Hz and SD is 0.067 Hz.

Thus, the way how to select optimal parameters of designed 2-order FLL-assisted 3-order PLL has been clearly explained through a 6-DoF trajectory model and the results of simulation show the designed system presents an excellent tracking performance in both phase (error less than 3°) and speed (error less than 0.1 Hz).

## 4. Error Analysis

From the analysis in chapter 2 and chapter 3, real-time estimation of roll angle is based on obtaining the phase information of sinusoidal signal induced by geomagnetic vector projecting on projectile in the rotary state through 2-order FLL-assisted 3-order PLL tracking system as well as the calculation of compensation angle. Therefore, the error of this method originates from the phase tracking loop, compensation angle, as well as blind area [22], in which the X-axis of the projectile is parallel to the geomagnetic vector and there is no projection on cross-section leading to the input of tracking loop is zero. Additionally, blind area has less effect on compensation angle for compensation angle is irrelevant with total intensity according to (5–7). All these three error sources will be discussed in this chapter.

### 4.1. Error Analysis of Tracking Loop

Measurement error of tracking loop is from both third-order PLL and second-order FLL. For a general PLL, the error resource mainly comes from dynamic stress error and phase jitter including thermal noise $\sigma_{\mathrm{tPLL}}$, oscillator jitter $\sigma_{v}$, and Allan deviation $\sigma_{A}$ related with the frequency of input signal [29].

For a third-order PLL, the dynamic stress error is given by

(22) $$\varphi_{d}^{p}=1/\omega_{\mathrm{np}}^{3}\left(d^{3}\varphi^{p}/dt^{3}\right)$$

where, $\varphi^{p}$ is the phase of $B_{Z}$ and in the circumstance of the projectile rotating in the form of acceleration, $d^{2}\varphi^{p}/dt^{2}$ is the phase acceleration of $B_{Z}$, and $d^{3}\varphi^{p}/dt^{3}=0$, corresponding to the analysis of steady-state error $e_{\mathrm{ss}}(\infty )$ in chapter 3.

Owing to frequency of $B_{Z}$ being no more than 400 Hz, phase error caused by $\sigma_{v}$ and $\sigma_{A}$ is too small so that they can be ignored. The thermal noise $\sigma_{t\mathrm{PLL}}$ can be expressed as

(23) $$\sigma_{\mathrm{tPLL}}=\frac{180{}^{\circ}}{\pi }\sqrt{\frac{B_{\mathrm{PLL}}}{C/N_{0}}(1+\frac{1}{2T_{\mathrm{roll}}C/N_{0}})}$$

where, $B_{\mathrm{PLL}}$ is the noise bandwidth, $C/N_{0}$ is carrier-to-noise ratio (Hz), and $T_{\mathrm{roll}}$ is integration time. According to (23), effects from $\sigma_{\mathrm{tPLL}}$ can be reduced by decreasing noise bandwidth $B_{\mathrm{PLL}}$ and increasing $C/N_{0}$ as well as $T_{\mathrm{roll}}$.

Thus, tracking error of PLL depends on the phase jitter caused by thermal noise $\sigma_{\mathrm{tPLL}}$ to a large degree. According to the rule of thumb for tracking threshold [29], only in the condition that 3-sigma jitter is no more than one fourth of the pull-in range of PLL discriminator could PLL track input signal constantly and stably. For example, for a four-quadrant arctangent discriminator, the pull-in range of phase discriminator is 360° and the tracking threshold of PLL should satisfy

(24) $$3\sigma_{\mathrm{tPLL}}\leq 90{}^{\circ}$$

Similarly, for a pure FLL, tracking error in this designed system mainly consists of frequency jitter caused by thermal noise $\sigma_{\mathrm{tFLL}}$ expressed as

(25) $$\sigma_{\mathrm{tFLL}}=\frac{1}{2\pi T_{\mathrm{roll}}}\sqrt{\frac{4FB_{\mathrm{FLL}}}{C/N_{0}}(1+\frac{1}{T_{\mathrm{roll}}C/N_{0}})}$$

where, F=1 when carrier-to-noise ratio $C/N_{0}$ reaches a high level and F=2 when the $C/N_{0}$ is at a low level and loop tracking works near to threshold. Based on the rule of thumb for tracking threshold mentioned above, $\sigma_{\mathrm{tFLL}}$ should satisfy

(26) 3σtFLL≤14Troll

In conclusion, the measurement error caused by PLL and FLL in this designed tracking loop mainly originates from phase jitter and frequency jitter in which thermal noise $\sigma_{t\mathrm{PLL}}$ and $\sigma_{t\mathrm{FLL}}$ account for the most. In order to eliminate this kind of error, the selection of parameters of noise bandwidth and integration time should meet the requirement that 3-sigma jitter is no more than one fourth of the pull-in range of discriminator.

### 4.2. Error From Compensation Angle

Based on analysis in chapter 2, and according to (1) and (4)–(7), the compensation angle ε is determined by geomagnetic information, inclination I and declination D, attitude of projectile, and pitch *θ* and yaw ψ (given by GPS receiver or other sensors in practice). In this part, they will be discussed.

Generally, the range of a howitzer is ~30 km, in which the declination and inclination changing slightly has little effect on the compensation angle except for some special locations such as the north pole and south pole.

Take some place $O_{L}$ in Beijing as a launching site, the declination and inclination of which are −6.8997° and 59.3551°, respectively. Based on the pitch angle of 6-DoF trajectory (shown in Figure 10b), azimuth at launch are set as 0°, 90°, 180°, and 270°, respectively. Then, compare the compensation angle based on original geomagnetic information of launching site $O_{L}$ and compensation angle based on updated information.

$N_{6}$, $E_{6}$, $S_{6}$, and $W_{6}$ are locations 30 km away from launching site in north, east, south, and west direction, respectively. In the north, geomagnetic information is updated at sites $N_{j}$ (j = 1, 2, 3, 4, 5), locations between $O_{L}$ and $N_{6}$, and they are 5 km from each other, and so are $E_{j}$ (j = 1, 2, 3, 4, 5), $S_{j}$ (j = 1, 2, 3, 4, 5), and $W_{j}$ (j = 1, 2, 3, 4, 5). All the data mentioned above come from [25,27,32,33].

The results of comparison of compensation angle are shown as follows.

**Figure 14:**
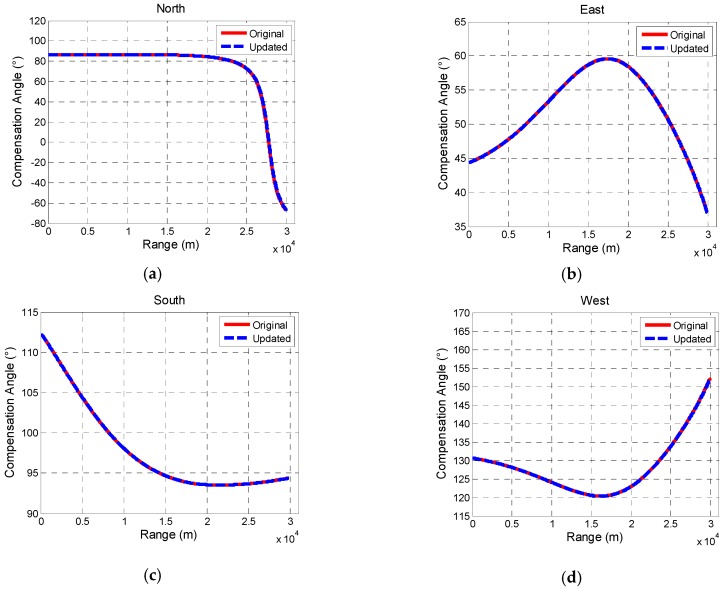
Comparison of compensation angle based on different geomagnetic information in north direction (**a**), east (**b**) direction, south (**c**) direction, and west (**d**) direction.

**Figure 15:**
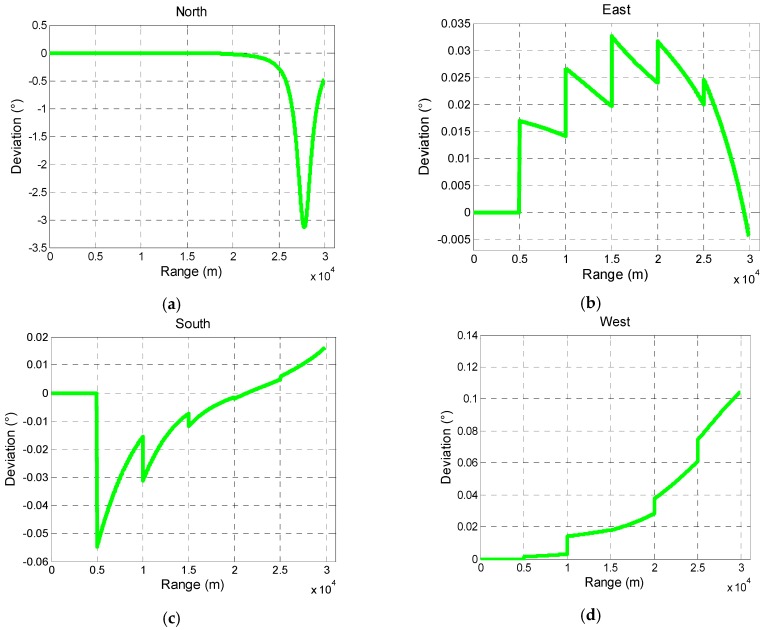
Deviation of compensation angle caused by different geomagnetic information in north direction (**a**), east (**b**) direction, south (**c**) direction, and west (**d**) direction.

Figure 14 and Figure 15 show that in the range of 30 km; the change of declination and inclination has little effect on the compensation angle. In the direction of east, south, and west, the deviation is within 0.15°. However, in the north direction, the compensation angle changes dramatically at the last 5 km which results in a comparatively large deviation 3.5°. It also indicates that deviation depends on the attitude of projectile in flight.

Analysis of deviation of compensation angle caused by different geomagnetic information is shown as follows.

Thus, if the accuracy of compensation angle cannot meet the requirements such as situation in north direction mentioned above, the information of geomagnetic field should be stored in advance and be exported to the tracking system to figure out compensation angle timely according to the location provided by GPS receiver. 

Yaw and pitch are given by GPS receiver (or by other sensors), and analysis of error from pitch and yaw is as follows.

Based on the attitude information from the 6-DoF trajectory mentioned above and the geomagnetic information of location $O_{L}$, compensation angle obtained from the attitude information with and without noise are to be compared in the circumstance of different yaw angles at the launch site.

**Figure 16:**
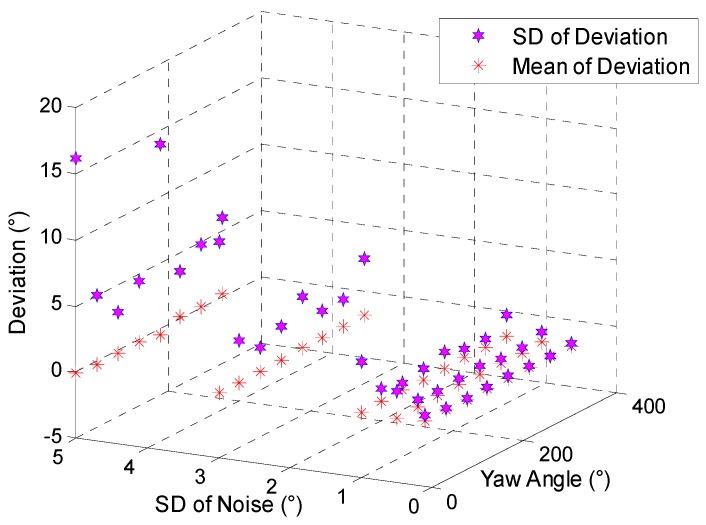
SD and mean of deviation of compensation angle caused by the noise.

Shown in Figure 16, SD and mean of deviation rise with the increasing SD of noise in each launching direction. This indicates that when the SD of noise of pitch and yaw is less than 5°, the mean and SD of deviation are approximately 0.5° and 6°, respectively. However, there are comparatively undesirable results in the north (yaw is 0°) and south (yaw is 180°) direction. When the SD of noise is 5° the SD of deviation reaches 16° and 14° in the north and south, respectively.

**Figure 17:**
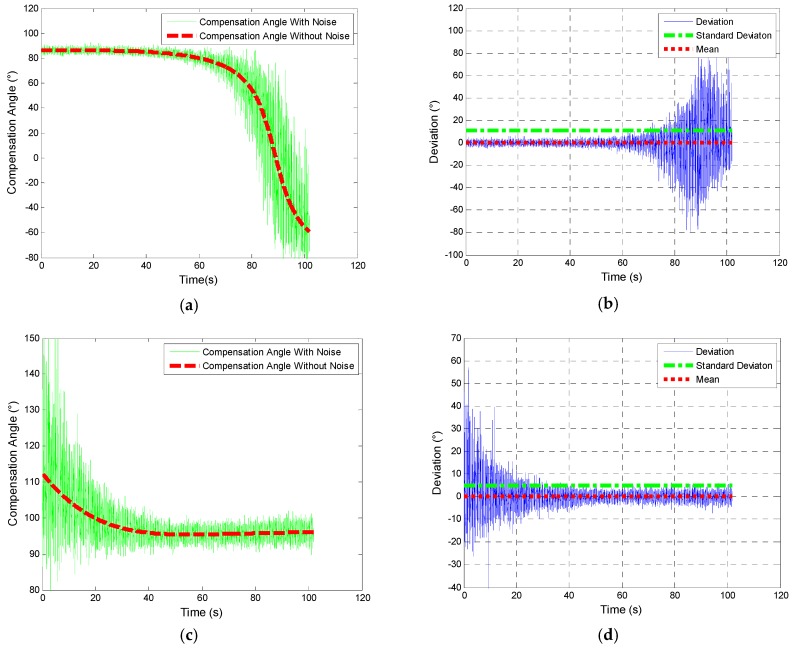
Compensation angle and deviation when yaw is 0° and 180°: (**a**,**c**) Description of compensation angle in the north and south direction and (**b**,**d**) description deviation of compensation angle.

Figure 17 describes the change of compensation angle and deviation of compensation angle when the SD of noise of yaw and pitch is 3°. When the yaw is 0° (shown in (a) and (b)), the deviation increases in the last 10 s; when the yaw is 180° (shown in (c) and (d)) the deviation is comparatively large at first and then converges till the end.

**Figure 18:**
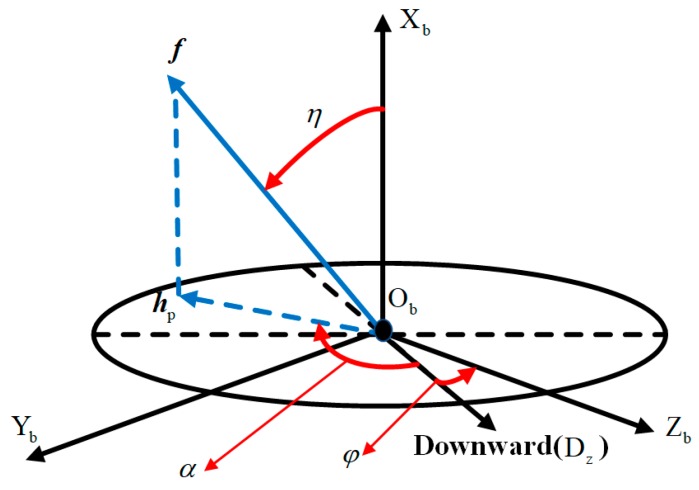
Cross-section and geomagnetic vector.

From Figure 10b, pitch ranges from 50° to −67°. Meanwhile, inclination is 59.263°. Thus, Figure 16 and Figure 17 indicate that when the body of projectile tends to be parallel to geomagnetic vector (shown in Figure 18), the compensation angle is influenced by the noise to a large degree. Moreover, for course correction is conducted at the terminal part of trajectory, large deviation in the previous part (when yaw is 180° shown in Figure 17a,b) has less effect on the guidance and control. In practice, attitude of projectile keeps changing in flight so that period of large deviation would not last as long as what is shown in Figure 17.

To conclude, in the range of howitzer, the change of geomagnetic information has less effect on the compensation angle. Deviation caused by it is less than 0.15°. Meanwhile, SD of noise from pitch and yaw should be less than 5° so that SD of deviation of compensation keep within 6°. Large deviation caused by certain azimuth will be discussed in detail next.

### 4.3. Error Caused by Geomagnetic Blind Area

The geomagnetic blind area is another factor determining the error of roll angle measurement. Shown in Figure 18, during the flight, when the X-axis is parallel to geomagnetic vector f, $B_{Z},$ the projection of f onto $Z_{b},$ is going to be zero or extremely small [22]. The range of the blind area depends on the location and attitude of projectile in flight.

Figure 18 describes the cross-section of projectile and the geomagnetic vector. $h_{p}$ is the projection of f on cross-section; η is the angle between X-axis and vector f; φ is the roll angle; and α is the angle between $h_{p}$ and downward direction (Z-axis of NED coordinate). Thus, $B_{Z}$ in (2.5) can be also described as

(27) $$B_{Z}=\left|f\right|\sin\eta \cos(\alpha +\varphi )$$

Referring to Zhang [24], there is a certain range of roll angle defined as the blind area of roll angle in which the detection module cannot satisfy the expected accuracy. It is determined by f, the total intensity of local field; Δφ, the expected precision of roll angle; η, the angle between X-axis and f; λ, resolution of magnetoresistive sensor selected. The blind area of roll angle is in the range of [24]

(28) $$\left|\sin(\alpha +\varphi )\right|<\frac{\lambda }{\Delta \gamma \left|f\right|\sin\eta }$$

in which the sensor is not able to detect the weak signals.

Thus, only when φ satisfies

(29) $$\left|\sin(\alpha +\varphi )\right|\geq \frac{\lambda }{\Delta \gamma \left|f\right|\sin\eta }$$

could the tracking loop obtain the rolling information satisfying the expected accuracy.

One can make a conclusion that to reduce the effects from blind area, in the selection of sensor, more consideration should be given to the resolution to reduce λ. Meanwhile, φ is determined by the attitude of projectile and local geomagnetic information. The period of blind area would not last too long for attitude keeps changing in flight. Furthermore, the tracking loop enables the measurement system to regain rolling information after tracking loop unlocked for the weak signals.

## 5. Experiments Validation on a Rotary Table

Experiments on a rotary table are carried out to verify the proposed method and analyze the accuracy. In this chapter, experiment scheme, the design of hardware, procedure, result, and analysis will be presented.

### 5.1. Description of Experiment

**Figure 19:**
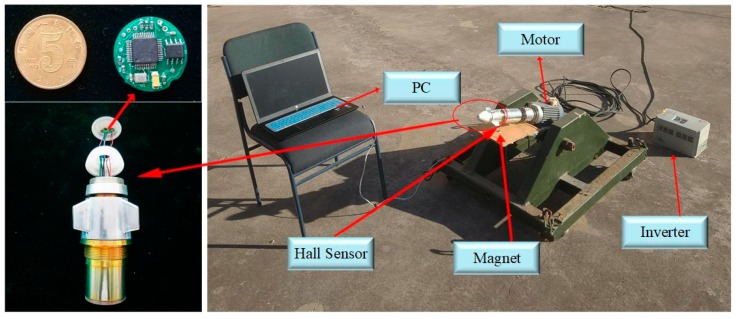
Experiments on rotary table.

The experiment is carried out on an open ground (the same position with [14]) which is shown in Figure 19.

The rotation of fake projectile is driven by motor controlled by inverter. Pitch and yaw is adjusted by the platform to simulate flight situation. There is a magnetoresistive sensor mounted at the top of fake projectile to detect the change of magnetic signals in rotary motion. Meanwhile, a hall sensor mounted on the side cylinder and a magnet mounted fixed below the cylinder, every time the hall sensor gets close to magnet (pointing to downward) in rotation it can produce a pulse to provide the reference to validate the phase information from tracking loop designed through magnetic signals.

Also, before rotation, magnetic and attitude information need to be initialized from PC through serial port. 

All those data created in the process during rotation is recorded and stored in memory module, which will be transmitted to PC by USB serial at last.

### 5.2. Design of Hardware

**Figure 20:**
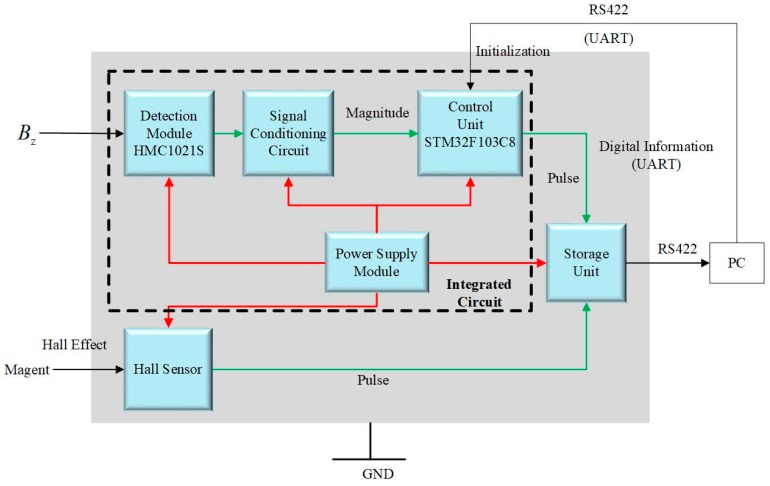
Design of integrated circuit.

Figure 20 shows the components of designed integrated circuit which consists of power supply module, detection module to detect magnetic signal, signal conditioning module, control unit, storage module, as well as hall sensor to provide reference.

The power module is designed to supply 8 V and 5 V DC voltage to other units. Magnetoresistive sensor HMC121S, produced by Honeywell, was applied to acquire the magnetic signals. The signal conditioning circuit consists of an amplifier, AD8227, which amplifies the output of detection module and realizes the shifting. The control unit is STM32F103C8T6 based on ARM Cortex-M produced by STMicroelectronics. Two 12-bit synchronized ADCs (Analog-to-Digital Converters) are embedded in that ship and the sampling frequency reaches to 1 MHz.

There are two chips to record digital information (such as roll angle, amplitude of $B_{Z}$, and speed) and analog information, pulse produced by a hall sensor and control unit, respectively. Storage of the former is 4 MB, and in the situation where sampling frequency is 1 kHz it is able to record data from controller continually for 33 min. The storage of the later is 2 MB, and in the situation where sampling frequency is 1 kHz it is able to record data continually for 33 min.

Meanwhile, the storage module is connected to PC by RS-422 serial port and data are analyzed by MATLAB in PC after every operation.

In practice, the hall sensor and magnetic sensor are mounted to point in the same direction. Every time hall sensor points to downward and get close to magnet it will produce a pulse which could be the reference of pulse produced by control unit for the tracking loop enables the system to produce a pulse every time the magnetic sensor points to downward. That is the way how this experiment verifies the accuracy of roll angle measurement.

### 5.3. Operation of Experiments

1. Clear the data in storage unit and initialize the magnetic and attitude information in controller. Start the storage unit.
2. Install the fake projectile in the rotary table and make sure that the magnetic sensor and hall sensor are pointing in the same direction. Turn on the integrated circuit and tracking loop.
3. Set a proper speed through inverter in advance. Turn on the motor and, simultaneously, record the time of rotary table starting to work as beginning time of experiment.
4. After motor working for a period of time, stop the motor and the acquisition of magnetic signals, as well as record the stop time. Read the data in PC from storage module through serial port.
5. Repeat the steps above and conduct another several groups of experiments.
6. Analyze the data and results.

### 5.4. Results and Analysis

Table 4 describes the initialization of magnetic and attitude information of every group of experiment. Magnetic information of each group is the same for they are conducted at the same place. Yaw angle is adjusted through rotary table to change the attitude of fake projectile to change experimental conditions. Speed is set by inverter and it is a nominal value.

Follow the steps in last section and optimize parameters of tracking loop through the way described in chapter 3 and results of experiments are as follows.

**Figure 21:**
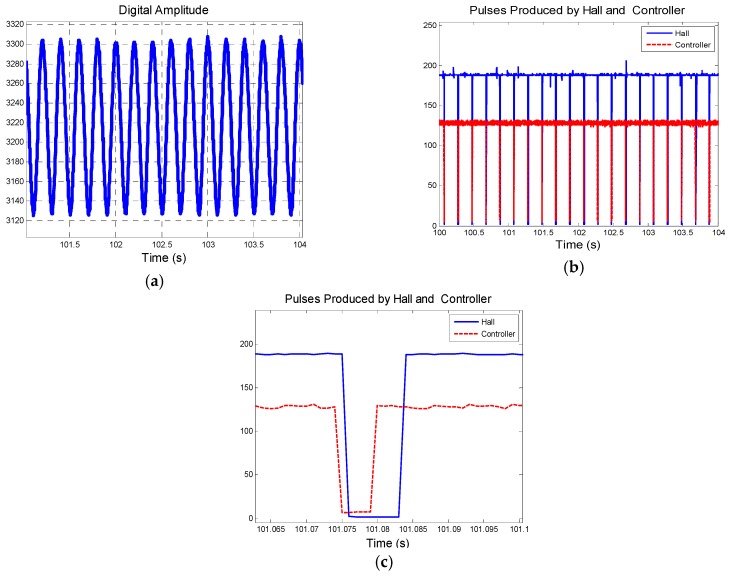

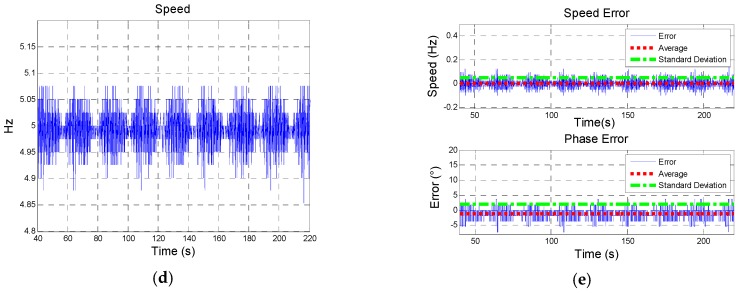
Results of group 1: (**a**) Description of digital amplitude of $B_{Z};$ (**b**) Comparison of pulses produced by hall sensor and controller and (**c**) is part of (**b**); (**d**) Description of speed figured out from controller; (**e**) Description of speed error and phase error.

Figure 21 shows the results of group 1 described in Table 4. (**a**) presents $B_{Z},$ the output of magnetic sensor in rotation. The start time is the 40th second and end time is the 220th second. (**c**) is part of figure (**b**), and they compare the pulses produced by hall sensor and controller, which is utilized to analyze phase error. Pulses produced by hall sensor are applied to figure out the actual rotational speed which also provides reference to the speed figured out from controller by tracking loop designed.

Figure (**d**) shows the speed figured out from controller and it is compared with speed figured from hall sensor and error analysis is shown in figure (**e**). It indicates that tracking loop designed is able to track the speed accurately and stably for the SD is 0.0469°.

Error analysis of phase is shown in figure (**e**). It indicates that the system designed presents a steady performance that SD is 1.9285°.

**Figure 22:**
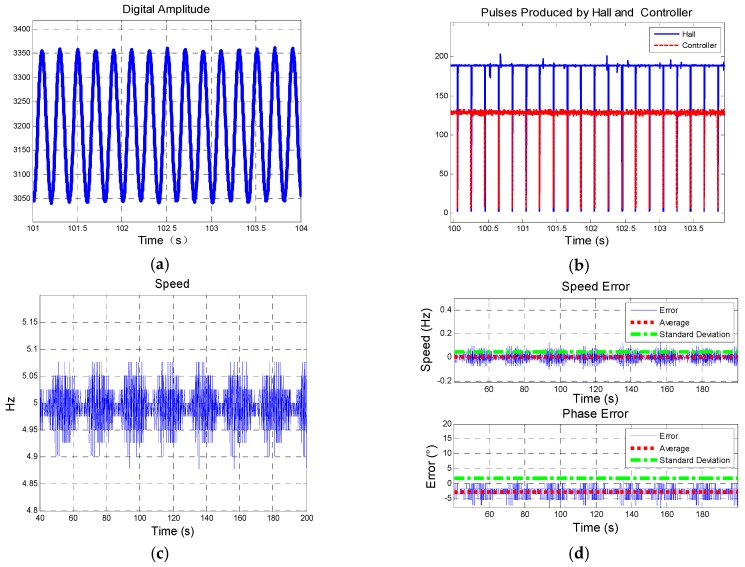
Results of group 2: (**a**) Description of digital amplitude of $B_{Z};$ (**b**) Comparison of pulses produced by hall sensor and controller; (**d**) Description of speed figured out from controller; (**e**) Description of speed error and phase error.

**Figure 23:**
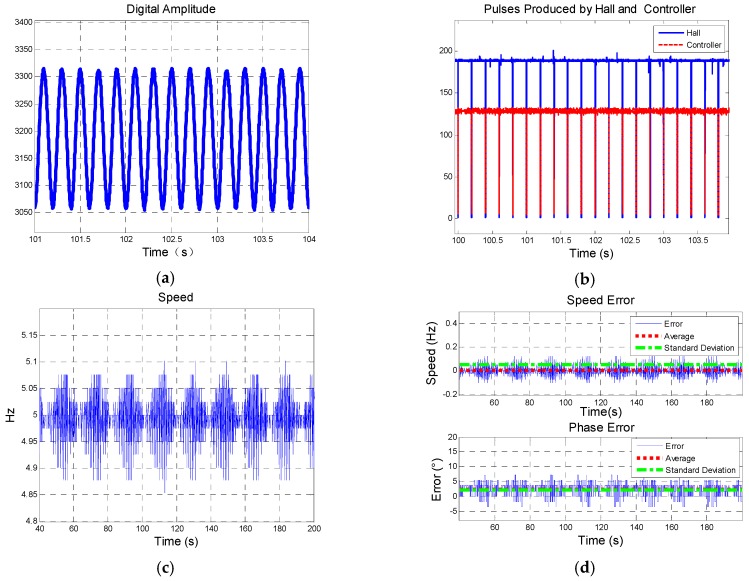
Results of group 3: (**a**) Description of digital amplitude of $B_{Z};$ (**b**) Comparison of pulses produced by hall sensor and controller; (**d**) Description of speed figured out from controller; (**e**) Description of speed error and phase error.

Similarly, Figure 22 and Figure 23 show the results of group 2 and group 3, respectively. Yaw angles in group 1, group 2, and group 3 are different, thus the digital amplitude varies in each group. The results are shown in Table 5.

Table 5 indicates that the tracking loop is able to track both phase and speed accurately and stably. SD of phase error is no more than 3°, which could reach the requirements of control and guidance module in course correction technology.

## 6. Conclusions

Real-time estimation of roll angle of spinning projectile based on phase-lock on signals from single-axis magnetometer is proposed and verified. This technology is based on information, position, velocity, yaw, and pitch from GPS receiver (or other sensors). Unlike the conventional measurement by inverse trigonometric operation based on amplitude of multiple outputs, single-axis magnetic sensor enables this system to measure rolling information without compensation based on a second-order frequency-locked loop (FLL) assisted third-order phase-locked loop (PLL).

A mathematical model based on coordinate transformation of the local North-East-Down (NED) coordinate system and body coordinate system was set up to analyze the relationship between roll angle and magnetic signal. There is a difference (compensation angle) between roll angle and phase information of magnetic signal detected. And obtaining the phase information and compensation angle is the key to figure out roll angle.

According to the practice, the spinning projectile rotates in the form of acceleration function, a second-order FLL-assisted third-order PLL is selected to track the phase information. Parameters of tracking loop depends on the frequency of input and are discussed to optimize system to keep excellent transient performance as well as accurate in different speed situation. A 6-DoF trajectory is given to verify the tracking system designed and results show an outstanding tracking performance in both phase (error less than 3°) and speed (error less than 0.1 Hz). 

Generally, error of roll angle measurement is required by guidance and control module. To eliminate the measurement error from tracking loop, the selection of parameters of noise bandwidth and integration time should meet the requirement that 3-sigma jitter is no more than one fourth of the pull-in range of discriminator. In the range of howitzer (~30 km), the change of declination and inclination has little effect on the compensation angle (deviation mostly keeps within 0.15°) and if necessary, the geomagnetic information should be stored in advance and exported to the tracking system to figure out compensation angle timely. Some filtering measurements should be taken to deal with attitude information from GPS receivers and error of yaw and pitch should be less than 5° so that SD of deviation of compensation angle mostly keeps within 6°. A magnetic sensor with a high resolution helps to eliminate error from blind area and the period of blind area would not last too long for attitude keeps changing in flight.

Experiments on rotary table are carried out to verify the proposed method. In the circumstance of nominal speed 5 Hz and attitude changed, the tracking loop is able to track both phase and speed accurately and stably. The mean of phase error is within 2.4° and SD of phase error is no more than 3°.

Profound research, such as improving accuracy and experiments with higher speed and changing attitude, will be conducted.

## Figures and Tables

**Table 1:** Influence of parameters on both accuracy and transient response.

Parameters	Performance	Performance	Performance
*B*_PLL_ 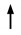	Tracking accuracy 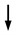	Transient Performance 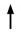	Steady-state Performance 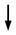
*ξ* 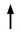	Cut-off Characteristics 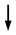	Transient Performance 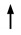	Steady-state Performance 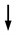
*B*_FLL_ 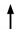	Cut-off Characteristics 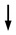	Transient Performance 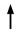	Steady-state Performance 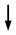

**Table 2:** Properties of projectile and field.

Properties of Projectile	Specifications	Properties of Geomagnetic Field	Specifications
Mass (kg)	46.88	Strength (mGs)	500
Width (m)	0.866	Declination (°)	−6.8285
Axial Inertial (kg·m^2^)	0.1658	Inclination (°)	59.263
Initial Attitude (°)	0 (Yaw); 51 (Pitch)	Sampling Frequency (Hz)	1000

**Table 3:** Optimized parameters.

Type of Projectile	Rotational Speed	Parameters of 3-Order PLL	Parameters of 2-Order FLL
155 mm Howitzer	300–134 Hz	$B_{\mathrm{PLL}}=$ 0.65	$B_{\mathrm{PLL}}=$ 0.7
		$T_{\mathrm{roll}}=$ 40 ms	ξ=0.707
			$T_{\mathrm{roll}}=$ 40 ms

**Table 4:** Parameters of experiments.

Group	Yaw	Pitch	Declination	Inclination	Nominal Speed	Duration
1	190°	18°	−6.8285°	56.2653°	5 Hz	40 s–220 s
2	280°	18°	−6.8285°	56.2653°	5 Hz	40 s–200 s
3	370°	18°	−6.8285°	56.2653°	5 Hz	40 s–200 s

**Table 5:** Results of experiments.

Group	Nominal Speed	Average (Phase Error)	SD (Speed Error)	SD (Phase Error)
1	5 r/s	1.487°	0.047°	1.929°
2	5 r/s	1.827°	0.045°	2.906°
3	5 r/s	2.320°	0.050°	2.684°
Average		1.878°	0.047°	2.506°

## References

[1] Carlucci D., Pellen R., Pritchard J., Demassi W. (2010). Smart Projectiles: Design Guidelines and Development Process Keys to Success.

[2] Mahajan C., Motghare V. (2010). Smart Munitions. Defence Sci J..

[3] Yuan W., Zhang J.Q., Yang F. (2015). Study on Stable Scanning of Terminal Sensitivity Projectile and Hardware-in-the-Loop Simulation System. Pure Appl. Math. J..

[4] Daso D.A. (2007). Weapons of Choice: The Development of Precision Guided Munitions (Review). Technol. Culture.

[5] Wang Z.F., Wang H. Target Location of Loitering Munitions Based on Image Matching. Proceedings of the 2011 6th IEEE Conference on Industrial Electronics and Applications.

[6] Liu H.W., Jiang C.L., Li M., Cheng X.Y. Weighted Self-Localization Algorithm of Networked Munitions. Proceedings of the 2013 International Conference on Information System and Engineering Management.

[7] Sonalkar R., James H. Communication Range Extension for the Intelligent Munitions System. Proceedings of the 2008 IEEE Military Communications Conference.

[8] Gagnon E., Marc L. Course Correction Fuze Concept Analysis for In-Service 155 mm Spin-Stabilized Gunnery Projectiles. Proceedings of the 2008 AIAA Guidance, Navigation and Control Conference and Exhibit.

[9] Perrin M. Course Correction Fuzes Integration Technologies. Proceedings of the 55th Annual Fuze Conference.

[10] Pettersson T., Buretta R., Cook D. Aerodynamics and Flight Stability for a Course Corrected Artillery Round. Proceedings of the 23rd International Symposium on BALLISTICS.

[11] Park H.Y., Kim K.J., Lee J.G., Park C.G. (2007). Roll Angle Estimation for Smart Munition. IFAC Proc. Vol..

[12] Harkins T.E., Wilson M.J. Measuring In-Flight Angular Motion with a Low-Cost Magnetometer. https://apps.dtic.mil/dtic/tr/fulltext/u2/a472265.pdf.

[13] Shen Q., Li M., Gong R. (2017). GPS Positioning Algorithm for a Spinning Vehicle with Discontinuous Signals Received by a Single-Patch Antenna. GPS Solutions.

[14] Deng Z.L., Shen Q., Deng Z.W. (2018). Roll Angle Measurement for a Spinning Vehicle Based on GPS Signals Received by a Single-Patch Antenna. Sensors.

[15] Changey S., Pecheur E., Bernard L., Sommer E., Wey P., Berner C. Real Time Estimation of Projectile Roll Angle Using Magnetometers: In-Flight Experimental Validation. Proceedings of the 2012 IEEE/ION Position, Location and Navigation Symposium.

[16] Changey S., Pecheur E., Brunner T. Attitude Estimation of a Projectile Using Magnetometers and Accelerometers: Experimental Validation. Proceedings of the IEEE/ION PLANS 2014.

[17] Harkins T.E., Davis B.S., Hepner D.J. (2001). Novel Onboard Sensor Systems for Making Angular Measurements on Spinning Projectiles, Aerospace/Defense Sensing, Simulation, and Controls. Acquisition, Tracking, and Pointing XV.

[18] Wilson M.J. (2004). Attitude Determination with Magnetometers for Gun-Launched Munitions.

[19] Changey S., Pecheur E., Wey P., Sommer E. (2013). Real-Time Estimation of Projectile Roll Angle Using Magnetometers: In-Lab Experimental Validation. Prog. Flight Dyn. Guidance Navig Control Fault Detect Avionics.

[20] Cao P., Yu J.Y., Wang X.M., Yao W.J., Wu Y.L. (2014). High-frequency Measurement and Calculation Study of Systematic Errors of High-rolling Projectile Roll Angle Based on a Combination of MR/GNSS. Acta Armamentarii.

[21] Cao P., Wang X.M., Yu J.Y., Wu Y.L. (2013). The Combined Measurement of Roll Angle of Projectile by Geomagnetic and Satellite and Error Analysis. http://en.cnki.com.cn/Article_en/CJFDTotal-DJZD201306043.htm.

[22] Shang J.Y., Deng Z.H., Fu M.Y., Wang S.T. (2016). A High-Spin Rate Measurement Method for Projectiles Using a Magnetoresistive Sensor Based on Time-Frequency Domain Analysis. Sensors.

[23] Wang Z., Gao F.Q., Gao M., Lu Z.C. Roll Angular Velocity Real-Time Tracking Algorithm Based on Geomagnetic Information with Frequency-Locked Loop. Proceedings of the 2nd International Conference on Civil, Materials and Environmental Sciences.

[24] Zhang H.B., Yang Y., Sun K. (2015). Calculation and Blind Area Analysis on Rolling Angle of Projectile Based on Geomagnetic Method. http://cpfd.cnki.com.cn/Article/CPFDTOTAL-DIDD201505003006.htm.

[25] International Geomagnetic Reference Field (IGRF-12). http://www.ngdc.noaa.gov/IAGA/vmod/.

[26] Cai G.W., Chen B.M., Lee T.H. (2011). Coordinate Systems and Transformations. Unmanned Rotorcraft Systems.

[27] NOAA The World Magnetic Model. https://www.ngdc.noaa.gov/geomag/WMM/DoDWMM.shtml.

[28] Gardner F.M. (2005). Introduction. Phaselock Techniques.

[29] Kaplan E.D., Christopher J.H. (2005). Satellite Signal Acquisition, Tracking, and Data Demodulation. Understanding GPS: Principles and Applications.

[30] Franklin G.F., Powell J.D., Emami-Naeini A. (2010). The Final Value Theorem. Feedback Control of Dynamic Systems.

[31] Rao P.P., Sutter B.M., Hong P.E. (1997). Six-Degree-of-Freedom Trajectory Targeting and Optimization for Titan Launch Vehicles. J. Spacecraft Rockets.

[32] Bai Du Map of Beijing. https://map.baidu.com/.

[33] GPSSPG Longitude and Latitude Based on Location. http://www.gpsspg.com/.

